# EBV-positive large B-cell lymphoma with an unusual intravascular presentation and associated haemophagocytic syndrome in an HIV-positive patient: report of a case expanding the spectrum of EBV-positive immunodeficiency-associated lymphoproliferative disorders

**DOI:** 10.1007/s00428-021-03142-1

**Published:** 2021-06-19

**Authors:** Luis Veloza, Chun-Yi Tsai, Bettina Bisig, Olivier Pantet, Lorenzo Alberio, Christine Sempoux, Matthias Cavassini, Laurence de Leval

**Affiliations:** 1 Institute of Pathology, Department of Laboratory Medicine and Pathology, Lausanne, University Hospital (CHUV) and Lausanne University (UNIL), Rue du Bugnon 25, CH-1011 Lausanne, Switzerland; 2 Service of Adult Intensive Care, Lausanne University Hospital (CHUV) and Lausanne University (UNIL), Lausanne, Switzerland; 3 Service and Central Laboratory of Hematology, Department of Oncology and Department of Laboratory Medicine and Pathology, Lausanne University Hospital (CHUV) and Lausanne University (UNIL), Lausanne, Switzerland; 4 Service of Infectious Diseases, Department of Medicine, Lausanne University Hospital (CHUV) and Lausanne University (UNIL), Lausanne, Switzerland

**Keywords:** Intravascular large B-cell lymphoma, EBV, Hepatic capillary haemangioma, HIV, Haemophagocytic syndrome

## Abstract

Intravascular large B-cell lymphoma is a rare and aggressive EBV-negative large B-cell lymphoma with a dismal outcome. Here, we describe the case of a 76-year-old HIV-positive patient with an acute presentation of systemic symptoms and rapidly fatal outcome. Autopsy revealed a disseminated large B-cell lymphoma with an intravascular distribution involving the liver, lymph nodes, spleen, and bone marrow and associated to fibrin thrombi in hepatic capillary haemangiomas. The neoplastic B cells (CD79a + / − , CD20 + / − , CD30 + , MUM1 + , PD-L1 +) showed a Hodgkin and Reed-Sternberg-like morphology and were EBV-positive with a latency type II (LMP1 + , EBNA2-). Haemophagocytosis was documented in the bone marrow and lymph nodes. This case illustrates the diagnostic challenges of large B-cell lymphoma with intravascular presentation. We found only five other cases of EBV-positive large B-cell lymphoma with an intravascular presentation in the literature, three of which had an underlying immunodeficiency adding to the broad spectrum of EBV-associated lymphoma in the setting of immunosuppression.

**Supplementary Information:**

The online version contains supplementary material available at 10.1007/s00428-021-03142-1.

## Introduction

Intravascular large B-cell lymphoma (IVLBCL) is a rare and aggressive B-cell neoplasia that affects mainly elderly patients, usually disseminated at diagnosis and characterized by the selective growth of neoplastic cells within the lumina of vessels, particularly the capillaries [1]. The disease usually affects individuals without underlying immunodeficiency and is Epstein-Barr virus (EBV)-negative [2]. Three variants of clinical presentation are currently recognized as follows: (1) a classical form (usually encountered in Western countries), which shows organ-related symptoms with frequent central nervous system and cutaneous involvement; (2) a haemophagocytic syndrome (HPS)-associated form (Asian variant) characterized by fever, hepatosplenomegaly, pancytopenia, and multiorgan failure; and (3) a cutaneous variant, associated with better outcome [2]. Here, we report a 76-year-old human immunodeficiency virus (HIV)-positive patient with a post-mortem diagnosis of EBV-positive large B-cell lymphoma with an unusual intravascular presentation. Interestingly, tumour cells invaded the lumina of hepatic capillary haemangiomas. We found five other cases of EBV-positive large B-cell lymphoma with an intravascular location in the literature. Collectively, these case reports emphasize the broad spectrum of EBV-associated lymphoproliferative disorders and a possible oncogenic role of EBV and immunosuppression in these rare instances.

## Case presentation

The patient was a 76-year-old Caucasian male known for HIV infection diagnosis 2 years ago in the Caribbean who started combined anti-retroviral therapy at diagnosis. The patient with recent onset of fatigue and 30 kg weight loss was admitted to the emergency department with altered general condition and fever (38 °C). He reported having interrupted the treatment for a couple of months. Abdominal sonography revealed hepatomegaly with some hypodense foci measuring up to 2 cm, consistent with hepatic haemangiomas. No lesion suspicious of neoplasia was observed. At admission, laboratory tests showed low CD4 + T-cell count, 83 cells/mm^3^; HIV viral load, 4.1 × 10^3^ copies/ml; Hb 92 g/l; leukocytes 4.4 × 10^9^/L with lymphopenia at 0.6 × 10^9^/L; platelets 113 × 10^9^/L; ferritin 8.033 µg/l (normal values: 24 to 336 µg/l); fibrinogen <1.5 g/L (2.0 to 4.0 g/L) and normal values of triglycerides (< 150 mg/dL). A high EBV viral load (400.000 copies/mL) suggested the possibility of a HPS, although in the course of the hospitalization only four of five criteria required for the diagnosis were met (fever, anaemia and thrombocytopenia, hypofibrinogenemia and high ferritin levels). Repeated haemocultures and multiple serologies remained negative. Despite antiretroviral therapy, broad-spectrum antibiotic and antifungal therapy, patient developed acute liver and renal failure, refractory vasoplegic shock, and died 5 days after admission.

The autopsy showed pericardial and pleural effusions, haemorrhagic ascites, splenomegaly (530 g), and hepatomegaly (2000 g). Two haemorrhagic-appearing lesions (2.4 and 3 cm) in the liver consistent with haemangiomas suspected by imaging studies were found. Small intra-abdominal and supra-diaphragmatic lymph nodes (< 1 cm) were identified.

**Fig. 1:**
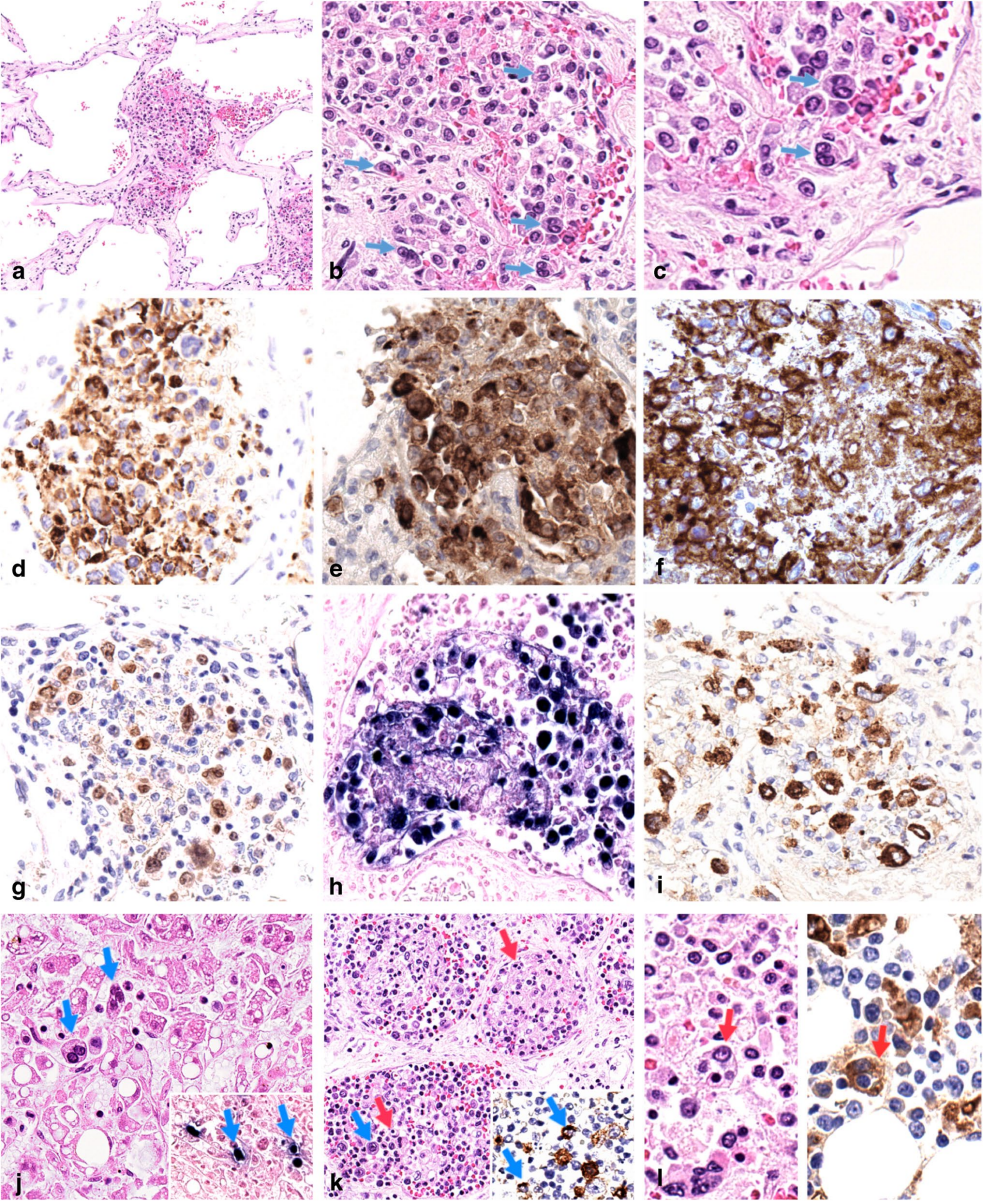
Histopathologic features of liver (**a–j**), lymph nodes (**k**), and bone marrow (**l**). Liver haemangioma showed aggregates of large tumour cells admixed to fibrin thrombi in the vascular lumina (**a**). The lymphoma cells were large pleomorphic sometimes resembling Reed-Sternberg cells (**b**, arrows, and **c**). The tumour cells were partially positive for CD20 (**d**), strongly positive for CD30 (**e**), PD-L1 (clone SP263) (**f**), and MUM1 (**g**). Neoplastic cells were positive for EBV (EBER-ISH) (**h**) and LMP1 (**i**). Atypical EBV-positive large tumour cells were identified in hepatic sinusoids (j, arrows; EBER-ISH, inset, arrows). Tumour cells were also observed colonizing lymph node’s blood vessels (**k**, blue arrow), with LMP1-expression (**k**, inset, blue arrows). Lymph node’s blood vessels (**k**) and bone marrow (**l**, left panel) comprised many histiocytes with engulfed red blood cells or nucleated cells (red arrows). CD68 (clone PG-M1) immunostaining highlights haemophagocytic histiocytes (**l**, right panel, red arrow). Original magnifications: **a** × 100, **b**, **d**, **e**, **f**, **g**, **h**, **i**, **j**, **l** × 400, **k** × 200, **c** × 600

Histologically, the liver lesions were typical of capillary haemangiomas and consisted of large vascular spaces lined by unremarkable endothelium. The lumina contained aggregates of large, atypical tumour cells with ample and pale cytoplasm, irregular nuclear contours, vesicular chromatin, some with prominent nucleoli or Reed-Sternberg-like morphology (Fig. 1a–c), often admixed with fibrin thrombi. The atypical large cells were partially positive for CD20 (Fig. 1d), and CD79a, strongly positive for CD30 (Fig. 1e) and PD-L1 (Fig. 1f) and partially expressed MUM1 (Fig. 1g). CD10, BCL6, CD138, LANA1 (HHV8), CD5, and MYC were negative. KI-67 proliferation index was around 60%. Virtually all tumour cells were positive for EBV by in situ hybridization with EBER probes (Fig. 1h) and by immunohistochemistry were LMP1-positive (Fig. 1i) and EBNA2-negative. Intravascular EBV-positive tumour cells were observed in the hepatic sinusoids outside the hepatic hemangioma (Fig. 1j), lymph nodes sinuses and small and medium-sized blood vessels (Fig. 1k), spleen, and bone marrow. Numerous histiocytes with abundant cytoplasm and ingested red blood cell, erythroid progenitors, or lymphocytes (haemophagocytosis) were identified in the lymph nodes’ blood vessels (Fig. 1k), liver, and bone marrow (Fig. 1l, left panel) highlighted by CD68 (Fig. 1l, right panel). No lesions of Kaposi’s sarcoma were observed. FISH studies using break-apart probes did not detect rearrangements of *BCL2*, *BCL6*, or *MYC*. High-throughput sequencing analysis using a customized panel of 54 genes relevant to the biology of mature B-cell lymphoma (Supplemental Information) did not detect mutations in *MYD88*, *CD79a*, or *EZH2*, genes recurrently altered in IVLBCL [2]. Multiple mutations suggestive of aberrant somatic hypermutation were detected at low variant allele frequency (3%) in the of *SOCS1* exon 2.

## Discussion

Here, we report a case of an EBV-positive large B-cell lymphoma with an unusual intravascular presentation, which manifested as a clinically fulminant disease in a deeply immunosuppressed patient with uncontrolled HIV infection. Similar to typical IVLBCL, the diagnosis was made post-mortem, highlighting the difficulties in reaching a timely diagnosis in these patients [1]. The HPS suspected clinically was convincingly confirmed by the histological findings at autopsy, and other characteristic findings of HPS-associated variant of IVLBCL were also observed [2], i.e., bone marrow involvement, fever, hepatosplenomegaly, and thrombocytopenia, which were present in our patient. Nevertheless, the HPS-associated variant of IVLBCL has been reported almost exclusively in Asian patients without associated immunosuppression or EBV infection [2, 3]. Therefore, it can be reasonably suggested that EBV infection may have played a role in the development of florid HPS in our patient.

We identified five other cases of EBV-positive large B-cell lymphoma with an unusual intravascular presentation reported in the literature, whose clinicopathological characteristics are shown in Table 1 [4–8]. These occurred in four men and one woman at a median age of 57 years (range 42–65 years). The four patients with documented ethnicity were Asian, in contrast to the Western origin of our case. Interestingly, three of the five patients had underlying immunosuppression (HIV in one case, immunosuppressive medication for autoimmune diseases in two cases) and localized disease was reported in three patients. With the exception of one case involving Kaposi’s sarcoma, EBV-positive neoplastic cells showed expression of B-cell markers with a non-CGB phenotype (CD10 − , MUM1 + , and BCL6 − / +), as our case. Three patients died between 1 and 12 months after diagnosis, one with HPS. Of note, the case reported in the HIV-positive patient involving vascular lumina of Kaposi sarcoma had a plasmablastic immunophenotype but was not tested for HHV8. In that peculiar setting, however, coinfection of the neoplastic B cells with HHV8 cannot be excluded.

**Table 1:** Clinicopathological features of EBV-positive large B-cell lymphoma with intravascular presentation

Author	Age (y)/sex Ethnicity	Immunodeficiency	Clinical symptoms	Involved organs	Laboratory findings	Imaging findings	Immunophenotypic/molecular findings	EBV/HHV8	HPS	Treatment	Outcome (months)
Hsiao CH et al. [4]	52/M Asian	HIV (2 CD4 + /mm^3^)	Cough, generalized purple maculopapular skin lesions, convulsions, hemiplegia	Autopsy: skin (within Kaposi sarcoma), brain (perivascular involvement)	NA	NA	CD45 + , CD3-, CD43-, CD20-, CD30-, CD56-, IG-	+ /NA	-	Not treated	DOD
Komeno et al. [5]	59/M Asian	Methotrexate/rheumatoid arthritis	Fever, loss of appetite, dyspnoea, dizziness	Biopsies: angiolipoma, bone marrow (interstitial and diffuse infiltration) Autopsy: CNS, adrenal glands	Pancytopenia, increased LDH	Infarctions in the cerebellum and the left lateral ventricle, splenomegaly, no lymphadenopathies	CD20 + , CD79a + , CD10-, Bcl6-, MUM1 + , CD5-, CD3-, CD56-	+ /NA	+	R-CHOP × 7, brain irradiation, intrathecal irradiation, cytarabine, prednisolone, salvage chemotherapy	DOD (12)
Tranchida et al. [6]	56/M NA	Yes, azathioprine/autoimmune hepatitis	Fever, disorientation, testicular pain	Testis	NA	Epididymal nodule	CD20 + , CD10 + , CD30 + , LMP1 +	+ /NA	-	Cessation of azathioprine, R-CHOP, radiotherapy	AWD (20)
Li Q et al. [7]	65/M Asian	No	Fever	Liver	Anaemia, thrombocytopenia increased LDH	Abnormal FDG uptake in liver, hepatosplenomegaly	CD20 + , PAX-5 + , MUM1 + , BCL6 + , CD5 + , CD3-, CD10- No *BCL6, BCL2*, or *MYC* rearrangements	+ / −	-	Antibiotics, dexamethasone, supportive treatment	DOD (1)
Yamada et al. [8]	42/F Asian	No	Fatigue, genital bleeding, weight loss, abdominal fullness	Incidental discovery in uterus, ovaries	Anaemia	Uterine leiomyomas, hydronephrosis	CD45 + , CD20 + CD79a + , lambda + , CD5 + , CD3-, CD10-, CD34-, cyclin D1-	+ /NA	-	R-CHOP × 6, high- dose etoposide	AWD (10)
Present case	76/M Caucasian	HIV (83 CD4 + /mm^3^)	Fatigue, weight loss, altered general condition, drowsiness, fever	Autopsy: liver, spleen, lymph nodes, bone marrow	Pancytopenia, high levels of AST, ALT, ferritin	Hepatic haemangioma, hepatomegaly, bilateral pleural effusions, cardiomegaly, no lymphadenopathies and splenomegaly	CD79a + / − , CD20 + / − , CD30 + , CD5-, MUM1 + , PD-L1 + No *BCL6, BCL2*, and *MYC* rearrangements *SOCS1* mutation	+ / −	+	Not treated	DOD (0.1)

*ALT* alanine aminotransferase, *AST* aspartate aminotransferase, *AWD* alive without disease, *DOD* died of disease, *EBV* Epstein-Barr virus, *F* female, *FDG* fluorodeoxyglucose, *HHV8* human herpes virus 8, *HIV* human immunodeficiency virus, *HPS* haemophagocytic syndrome, *IG* immunoglobulin, *LDH* lactate dehydrogenase, *M* male, *NA* no data available, *R-CHOP* rituximab, cyclophosphamide, doxorubicin, vincristine, prednisone

To this regard, there are few case reports of HHV8-positive large B-cell lymphoma with intravascular presentation, all occurring in immunosuppressed patients (HIV and organ transplantation) [9–15]. Coexistence of HHV8-positive large B-cell lymphoma with intravascular presentation with Kaposi sarcoma, both disseminated and limited to the skin were described frequently in those cases, as well as co-infection of tumour cells by EBV (62%). Those cases showed a plasmablastic morphology and immunophenotype (CD20-, MUM1 +). Diagnosis was made at post-mortem examination and all patients rapidly succumbed 2 months or less after of admission to the hospital. Interestingly, cases of polyclonal HHV8-positive circulating plasmablastic cells IgM *λ* have been described in HIV-positive patients and severe symptoms of muticentric Castleman disease, which may mimick plasmablastic leukemia/lymphoma [16].

The fact that EBV and/or HHV8-positive large B-cell lymphoma with intravascular presentation show varied clinical and pathological presentations, heterogenous outcome and occur in association with immunosuppresion, suggests that these cases are distinct from IVLBCL and rather represent virus-associated large B-cell lymphoma with an unusual intravascular location. Table 2 summarizes the main clinicopathological features of IVLBCL and the virus-associated large B-cells lymphoma with unusual intravascular location.

**Table 2:** Differential diagnosis of large B-cell lymphomas with intravascular presentation

	IVLBL classic variant	IVLBL HPS–associated variant	IVLBL cutaneous variant	EBV + large B-cell lymphoma with intravascular presentation [Ref. 4–8]	HHV8 + , EBV + / − large B-cell lymphoma with intravascular presentation [9–15]
Median age	67 years	67 years	59 years	57 years	39 years
Gender	M = F	M = F	F > M	M > F	M > F
Ethnicity	Western	Asian	Western	Mostly Asian	Western
Immunodeficiency association	No	No	No	HIV or immunosuppressive treatment for autoimmune disorders	HIV, post-transplant setting
Clinical presentation	Fever, organ-specific local symptoms, CNS and cutaneous involvement, B symptoms, multiorgan failure	Multiorgan failure, hepatosplenomegaly, pancytopenia	Single or multiple lesions of the skin with negative systemic staging	Fever, fatigue, weight loss, organ-specific local symptoms	Fever, weight loss, Kaposi sarcoma lesions, hepatosplenomegaly, pleural effusions
Organs involved	Widely disseminated, frequent SNC and skin involvement	Bone marrow, liver, spleen	Skin	Variable, localized disease (50%)	Spleen, liver, pleural cavities, skin
Laboratory findings	Anaemia, increased LDH	Anaemia, thrombocytopenia, increased LDH	Normal leukocyte and platelet counts	Anaemia, pancytopenia	Pancytopenia, anaemia, thrombocytopenia
Cytology/ Immunophenotype	Large, atypical cells with prominent nucleoli/ CD20 + , MUM1 + , BCL6-/ + , CD10-/ + , CD5 + (38%)			Large, atypical cells with prominent nucleoli / CD20 + , MUM1 + , BCL6-/ + , CD10-/ + , CD5 + (50%)	Large, atypical cells with plasmablastic morphology/ CD45 + , CD20-, MUM1 +
HPS	-	+	-	+ (33%) or -	-
EBV/HHV8	-/-	-/-	-/-	+ / −	+ (62%)/ +

*CNS* central nervous system, *EBV* Epstein-Barr virus, *F* female, *HHV8* human herpes virus 8, *HIV* human immunodeficiency virus, *HPS* haemophagocytic syndrome, *IVLBL* intravascular large B-cell lymphoma, *LDH* lactate dehydrogenase, *M* male

Other distinctive morphological features in our case were the markedly pleomorphic cytomorphology including Reed-Sternberg-like cells, and the immunophenotype characterized by an attenuated B-cell program, and strong CD30 and PD-L1 expression. To this regard, rare cases of EBV-positive, HHV8-positive large B-cell lymphoma with Hodgkin/Reed-Sternberg-like morphology have been reported, interestingly in HIV-negative patients, but not with an intravascular distribution [9, 17].

This case represents the first report of an EBV-positive large B-cell lymphoma colonizing hepatic haemangiomas. Interestingly, the lymphoma cells were mostly present in association with fibrinous thrombi partially filling the large angiomatous spaces. On a purely histopathological basis, these findings are reminiscent of fibrin-associated diffuse large B-cell lymphoma [1]. In this rare form of large B-cell lymphoma, EBV-positive tumour cells show a latency III program and do not form tumour masses [1]. This B-cell proliferation develops within fibrinous deposits in the walls of pseudocysts, in the cardiovascular system, in cavities, in association with prostheses, or in haematomas [1]. However, in contrast to fibrin-associated diffuse large B-cell lymphoma, our patient presented a profound immunosuppression with a disseminated disease, an EBV-associated HPS and an aggressive clinical course, suggesting that viral oncogenic mechanisms, including latent protein expression (such as LMP1), were determinant for the pathogenesis and clinical course of the disease.

In conclusion, large B-cell lymphoma with intravascular presentation, including IVLBCL, remains a diagnostic and therapeutic challenge due to nonspecific and acute clinical presentation, frequently hindering an early and accurate diagnosis. Those cases associated with viral infection, such as EBV and/or HHV8, present distinct clinical-pathological features and develop almost invariably in the setting of immunosuppression. These findings suggest they correspond to an unusual presentation of immunodeficiency-associated B-cell lymphoproliferative disorders, expanding the spectrum of EBV-positive large B-cell lymphoma.

## Supplementary Information

Below is the link to the electronic supplementary material.

Supplementary file1 (DOCX 17 KB)
