# Supplementary material for: EBV-positive large B-cell lymphoma with an unusual intravascular presentation and associated haemophagocytic syndrome in an HIV-positive patient: report of a case expanding the spectrum of EBV-positive immunodeficiency-associated lymphoproliferative disorders
Source: Virchows Arch. 2021 Jun 19;480(3):699–705. doi: 10.1007/s00428-021-03142-1 (PMC8989855; doi:10.1007/s00428-021-03142-1)
Supplement: Supplementary file 1 — Supplementary file1 (DOCX 17 KB) [file 428_2021_3142_MOESM1_ESM.docx]

Virchows Archiv

**EBV-positive large B-cell lymphoma with an unusual intravascular presentation and associated haemophagocytic syndrome in a HIV-positive patient: report of a case expanding the spectrum of EBV-positive immunodeficiency-associated lymphoproliferative disorders**

Veloza Luis*, Tsai Chun-Yi*, Bisig Bettina*, Pantet Olivier†, Alberio Lorenzo §, Sempoux Christine*, Cavassini Matthias #, de Leval Laurence*‎

* Institute of Pathology, Department of Laboratory Medicine and Pathology, Lausanne University Hospital (CHUV) and Lausanne University (UNIL), Lausanne, Switzerland.

† Service of Adult Intensive Care, Lausanne University Hospital (CHUV) and Lausanne University (UNIL), Lausanne, Switzerland.

§ Service and Central Laboratory of Hematology, Department of Oncology and Department of Laboratory Medicine and Pathology Lausanne University Hospital (CHUV) and Lausanne University (UNIL), Lausanne, Switzerland.

# Service of Infectious Diseases, Department of Medicine, Lausanne University

Hospital (CHUV) and Lausanne University (UNIL), Lausanne, Switzerland.

Corresponding author: Laurence de Leval MD PhD

**Supplemental methods**

High-throughput sequencing analysis was performed using a customized panel, which covers 54 genes relevant to the biology of mature B-cell lymphoma (*ARID1A, ATM, B2M, BCL10, BCL2, BCL6, BIRC3, BRAF, BTK, CARD11, CCND1, CCND3, CD274 [PDL1], CD58, CD79A, CD79B, CDK4, CDKN2A, CIITA, CREBBP, CXCR4, EP300, EZH2, FOXO1, GNA13, ID3, IRF4, KLF2, KMT2C, KMT2D, MAP2K1, MAP3K14, MEF2B, MYC, MYD88, NFKBIE, NOTCH1, NOTCH2, PDCD1LG2 [PDL2], PIK3CD, PLCG2, PRDM1, PTEN, PTPRD, REL, SF3B1, SOCS1, STAT6, TCF3, TNFAIP3, TNFRSF14, TP53, TRAF2, XPO1*). Briefly, 100 to 200 ng (Qubit) of DNA template was used to prepare DNA libraries with the KAPA HyperPlus library preparation kit (Roche, Pleasanton, CA). Target enrichment of the DNA libraries was performed by hybridization capture with a custom design of xGen Lockdown Probes (Integrated DNA Technologies, Coralville, IA) covering the full coding sequences of the targeted genes. Enriched libraries were sequenced on a MiSeq™ System (Illumina, San Diego, CA). Sequence analysis was based on established algorithms and pipelines according to GATK best practices (The Genome Analysis Toolkit) standards. Briefly, forward and reverse reads were aligned to the human genome (GATK repository, build 37 decoy) using a BWA aligner (v0.7.5a). BAM files were subjected to PCR duplicate removal (Picard v1.119), followed by realignment around indels and base recalibration using GATK tools (v3.7). Single-nucleotide and indel variant calling was performed using both samtools mpileup (v1.2) and VarScan (v2.3.7), and MuTect2 algorithm (GATK v3.7). The union of the variant calls were annotated for presence in dbSNP and COSMIC databases and mutation effect on gene transcript by SnpEff (v.4.3). Further variant filtering was carried out in R, keeping variants that showed an allele frequency >1%, having at least 50 reads supporting the reference sequence and >5 reads supporting the variant. A filter based on a list of known artifacts was also applied. All retained alterations were confirmed by visual inspection with the Integrative Genomics Viewer (IGV) tool.
